# Supplementary material for: Pathological C-terminal phosphomimetic substitutions alter the mechanism of liquid-liquid phase separation of TDP-43 low complexity domain
Source: bioRxiv. 2024 Mar 27:2024.03.21.586202. Preprint. [Version 1] doi: 10.1101/2024.03.21.586202 (PMC10996529; doi:10.1101/2024.03.21.586202)
Supplement: 1 [file NIHPP2024.03.21.586202V1-supplement-1.pdf]

bioRxiv preprint doi: https://doi.org/10.1101/2024.03.21.586202; this version posted March 27, 2024. The copyright holder for this preprint (which was not certified by peer review) is the author/funder, who has granted bioRxiv a license to display the preprint in perpetuity. It is made available under aCC-BY 4.0 International license.

Supplementary Information

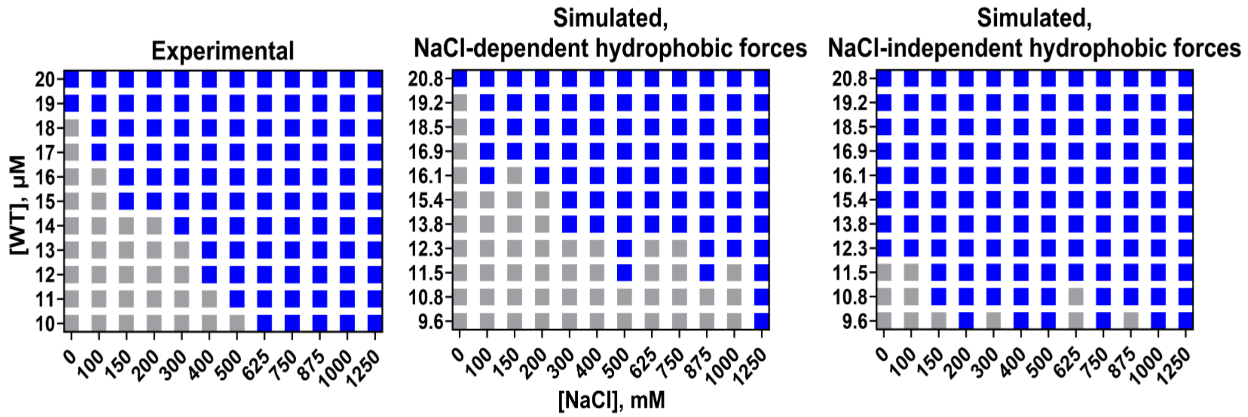

Supplementary Figure S1. Simulations with explicit NaCl-dependent hydrophobic forces reproduce experimental trends more accurately than NaCl-independent hydrophobic forces. WT protein phase diagrams generated from experimental data (*Left*), simulations with NaCl-dependent hydrophobic forces (*Middle*), and simulations with NaCl-independent hydrophobic forces (*Right*). Experiments and simulations were done at room temperature in 20 mM potassium phosphate buffer, pH 7.4.

bioRxiv preprint doi: https://doi.org/10.1101/2024.03.21.586202; this version posted March 27, 2024. The copyright holder for this preprint (which was not certified by peer review) is the author/funder, who has granted bioRxiv a license to display the preprint in perpetuity. It is made available under aCC-BY 4.0 International license.

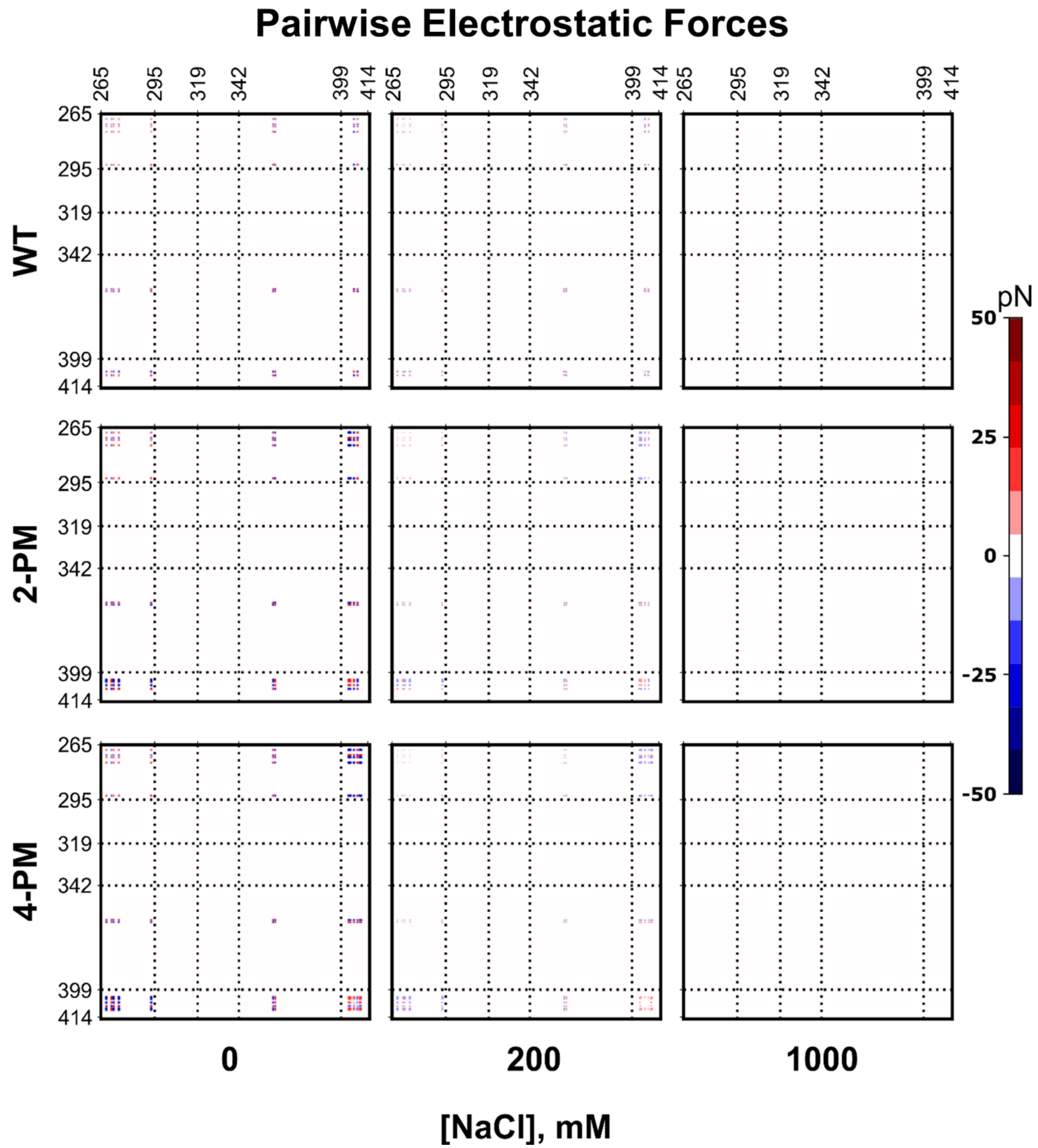

Supplementary Figure S2. Intermolecular residue-residue pairwise electrostatic force plots of phase-separated TDP-43 LCD variants. Pairwise intermolecular electrostatic forces of WT TDP-43 LCD (*Top*), 2-PM TDP-43 LCD (*Middle*) and 4-PM TDP-43 LCD (*Bottom*) at various ionic strengths. Data were generated from 20.8 µM protein simulations.

bioRxiv preprint doi: https://doi.org/10.1101/2024.03.21.586202; this version posted March 27, 2024. The copyright holder for this preprint (which was not certified by peer review) is the author/funder, who has granted bioRxiv a license to display the preprint in perpetuity. It is made available under aCC-BY 4.0 International license.

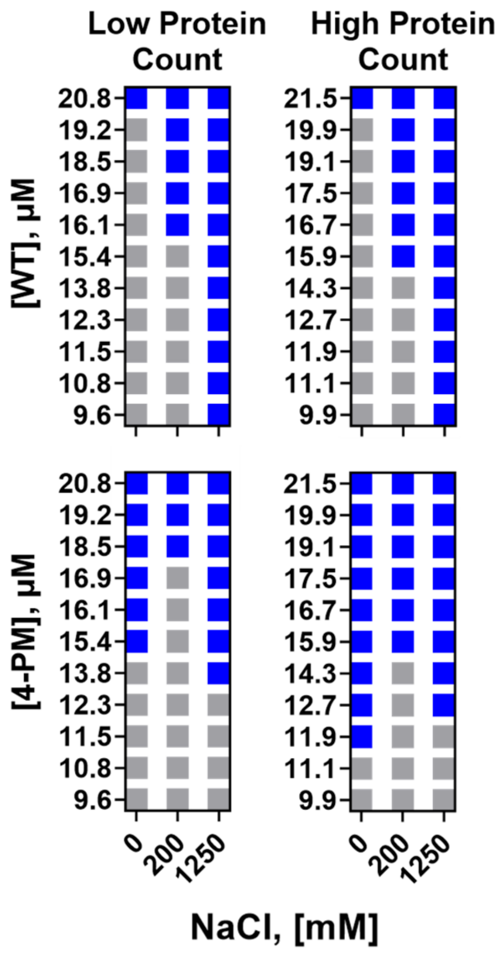

Supplementary Figure S3. Low protein count simulation results do not substantially differ from high protein count simulation results. Phase diagrams generated from low protein count simulations (*Left*), and high (5x) protein count simulations (*Right*) for WT protein (*Top*) and 4-PM protein (*Bottom*). Simulations were done at room temperature in 20 mM potassium phosphate, pH 7.4.

bioRxiv preprint doi: https://doi.org/10.1101/2024.03.21.586202; this version posted March 27, 2024. The copyright holder for this preprint (which was not certified by peer review) is the author/funder, who has granted bioRxiv a license to display the preprint in perpetuity. It is made available under aCC-BY 4.0 International license.

Amino acid	Mass m [amu]	Charge q [e]	Hydrophobicity λ	Size σ [Å]
ALA	71.08	0	0.730	5.04
ARG	156.2	1	0	6.56
ASN	114.1	0	0.432	5.68
ASP	115.1	-1	0.378	5.58
CYS	103.1	0	0.595	5.48
GLN	128.1	0	0.514	6.02
GLU	129.1	-1	0.459	5.92
GLY	57.05	0	0.649	4.50
HIS	137.1	0.5	0.514	6.08
ILE	113.2	0	0.973	6.18
LEU	113.2	0	0.973	6.18
LYS	128.2	1	0.514	6.36
MET	131.2	0	0.838	6.18
PHE	147.2	0	1	6.36
PRO	97.12	0	1	5.56
SER	87.08	0	0.595	5.18
THR	101.1	0	0.676	5.62
TRP	186.2	0	0.946	6.78
TYR	163.2	0	0.865	6.46
VAL	99.07	0	0.892	5.86

Supplementary Table S1. Amino acid parameters for coarse-grained simulations. The fundamental properties used for simulations of each of the 20 amino acids. From Dignon et al. 2018.
